# autoRasch: An R Package to Do Semi-Automated Rasch Analysis

**DOI:** 10.1177/01466216221125178

**Published:** 2022-10-10

**Authors:** Feri Wijayanto, Ioan Gabriel Bucur, Perry Groot, Tom Heskes

**Affiliations:** 1 Institute for Computing and Information Sciences, 6029 Radboud University, Nijmegen, The Netherlands; 2 Department of Informatics, Universitas Islam Indonesia, Indonesia

**Keywords:** Rasch analysis, semi-automated analysis, PJMLE, coordinate descent, lasso penalty

## Abstract

The R package autoRasch has been developed to perform a Rasch analysis in a (semi-)automated way. The automated part of the analysis is achieved by optimizing the so-called *in-plus-out-of-questionnaire log-likelihood* (IPOQ-LL) or IPOQ-LL-DIF when differential item functioning (DIF) is included. These criteria measure the quality of fit on a pre-collected survey, depending on which items are included in the final instrument. To compute these criteria, autoRasch fits the generalized partial credit model (GPCM) or the generalized partial credit model with differential item functioning (GPCM-DIF) using penalized joint maximum likelihood estimation (PJMLE). The package further allows the user to reevaluate the output of the automated method and use it as a basis for performing a manual Rasch analysis and provides standard statistics of Rasch analyses (e.g., outfit, infit, person separation reliability, and residual correlation) to support the model reevaluation.

## Description of the Package

Rasch analysis is a popular statistical method for developing and validating instruments to measure psychological aspects of humans. Typically, this method is done manually by experts with the assistance of software packages such as WINSTEP, RUMM2030, ConQuest, or eRM (www.rasch.org, 2014). Initially, the responses from an original survey need to be fitted to the model, either with the Rasch model (Rasch, 1960) in dichotomous cases or with the partial credit model (PCM) (Masters, 1982) in polytomous cases. As Rasch properties are checked (e.g., item goodness-of-fit, local dependency, reliability, and unidimensionality), items that misfit are removed one by one manually in an iterative process (Mul et al., 2021; O’Brien et al., 2021). Therefore, this procedure can be relatively time-consuming, especially when dealing with large and complex datasets. In addition, since the decisions on which items to include are partly based on human judgments combined with clinical expertise, different experts often select instruments that are different but equally suited.

The R (R Core Team, 2021) package autoRasch is a new tool for performing Rasch analysis in a (semi-)automatic manner. This package implements novel criteria that have been shown to naturally incorporate the standard properties of Rasch analysis (e.g., item goodness-of-fit, local dependency, reliability, unidimensionality, and differential item functioning). These criteria are called the *in-plus-out-of-questionnaire log-likelihood with differential item functioning* (IPOQ-LL-DIF) for cases incorporating DIF and *in-plus-out-of-questionnaire log-likelihood* (IPOQ-LL) for non-DIF cases (Wijayanto et al., 2021, 2022).

The IPOQ-LL-DIF and IPOQ-LL are computed by implementing the generalized partial credit model with differential item functioning (GPCM-DIF) (Wijayanto et al., 2022) and the generalized partial credit model (GPCM) (Muraki, 1992), respectively. For simplicity, the autoRasch package implements penalized joint maximum likelihood estimation (PJMLE) to fit these models. Moreover, recent studies have shown that PJMLE could yield comparable estimates to the marginal maximum likelihood estimation (MMLE) (Chen et al., 2019; Paolino, 2013; Robitzsch, 2021).

In contrast to the standard Rasch analysis, which investigates and removes items in a manual iterative way using statistics and expert knowledge, the autoRasch package attempts to automate the process by maximizing either the IPOQ-LL-DIF or IPOQ-LL scoring criteria. To maximize the score, this package implements a stepwise selection search method. The output of the stepwise selection search is formatted in a way that makes it easy to read, and it contains relevant information (e.g., the highest score and the items that yield it). Additionally, the change in score when sequentially removing items can be observed visually.

As a semi-automated method, our new procedure always welcomes the application of expert knowledge, for example, through pre- and post-analysis. To facilitate these pre- and post-analyses, the autoRasch package implements the fitting functions for the partial credit model (PCM) (Masters, 1982) and the partial credit model with differential functioning (PCM-DIF) (Wijayanto et al., 2022). Some standard Rasch statistics (e.g., outfit and infit statistics (Wright & Masters, 1982), residual correlation (Marais, 2013), and person/item separation reliability (PSR/ISR) (Wright & Masters, 1982) are also implemented. In addition, some typical graphical tools are also available (e.g., person-item map, item characteristic curve (ICC), and expected value curve (EVC)).

## Availability, Documentation, and Distribution

The autoRasch package, user’s manual, and sample codes are available from Comprehensive R Archive Network (CRAN) repository at https://CRAN.R-project.org/package=autoRasch. The development version of the package is hosted on GitHub (https://github.com/fwijayanto/autoRasch).

## Supplemental Material

Supplemental Material - autoRasch: An R Package to Do Semi-Automated Rasch Analysis Click here for additional data file. Supplemental Material for autoRasch: An R Package to Do Semi-Automated Rasch Analysis by Feri Wijayanto, Ioan Gabriel Bucur, Perry Groot, and Tom Heskes in Applied Psychological Measurement
