# Supplementary material for: autoRasch: An R Package to Do Semi-Automated Rasch Analysis
Source: Appl Psychol Meas. 2022 Oct 10;47(1):83–5. doi: 10.1177/01466216221125178 (PMC9679921; doi:10.1177/01466216221125178)
Supplement: Supplemental Material - autoRasch: An R Package to Do Semi-Automated Rasch Analysis [file sj-pdf-1-apm-10.1177_01466216221125178.pdf]

autoRasch: An R Package to do Semi-automated Rasch Analysis

Feri Wijayanto
Radboud University
and Universitas Islam Indonesia

Ioan Gabriel Bucur
Radboud University

Perry Groot
Radboud University

Tom Heskes
Radboud University

Abstract

The R package **autoRasch** has been purposefully developed to perform a Rasch analysis in a (semi-)automated way. The automated part of the analysis is achieved by optimizing the so-called *in-plus-out of questionnaire log likelihood* (IPOQ-LL) or IPOQ-LL-DIF when differential item functioning is included. These criteria measure the quality of fit on a pre-collected survey, depending on which items are included in the final instrument. To compute these criteria, **autoRasch** fits the partial credit model (PCM), the partial credit model with differential item functioning (PCM-DIF), the generalized partial credit model (GPCM), or the generalized partial credit model with differential item functioning (GPCM-DIF) using penalized joint maximum likelihood estimation (PJMLE). The package further allows the user to reevaluate the output of the automated method and use it as a basis for performing a manual Rasch analysis, and provides standard statistics of Rasch analyses (e.g., outfit, infit, person separation reliability, and residual correlation) to support the model reevaluation.

Keywords: Rasch analysis, semi-automated analysis, PJMLE, coordinate descent, lasso penalty.

1. Introduction

A popular way to validate a questionnaire and to measure one's abilities given subjects' responses is the Rasch model (Rasch 1966) and its family. With the help of various kinds of software (e.g., RUMM, Winstep) and packages (e.g., eRm, ltm) listed in `https://www.rasch.org/software.htm`, Rasch analysis is typically done in a manual iterative manner. Using the result of standard statistics (e.g., outfit, infit, local dependency, reliability, unidimensionality), misfit items are removed and the remaining items are then reevaluated until a psychometrically optimal set of items is obtained. Nevertheless, which statistics to begin with, which items need to be removed first, and when to stop are decisions to be made by experts. Consequently, this procedure is, undoubtedly, a laborious task that somewhat relies on expertise and human judgment. Moreover, different experts often favor non-identical but equally befitting items.

This article introduces the **autoRasch** package as a new tool for performing Rasch analyses in

a (semi-)automatic manner. This package implements novel criteria that have been shown to naturally incorporate the standard properties of Rasch analyses. These criteria are called the in-plus-out-of-questionnaire log likelihood with differential item functioning (IPOQ-LL-DIF) for DIF incorporated cases and in-plus-out-of-questionnaire log-likelihood (IPOQ-LL) for non-DIF cases (Wijayanto, Bucur, Mul, Groot, van Engelen, and Heskes 2022; Wijayanto, Mul, Groot, van Engelen, and Heskes 2021). Both criteria are developed based on the Rasch model by considering the discrimination and differential functioning over items, which will be discussed in Section 2. Section 2 also explains the techniques for estimating the models and how they differ from what some other packages offer. The formulation of the criteria is then discussed in Section 3 together with the implemented optimization procedure.

As mentioned, **autoRasch** aims to automate the iterative process of a Rasch analysis as much as possible. In spite of that, as in any statistical analysis, the final results have to be treated and analyzed carefully to judge whether they satisfy the modeler's desiderata. Therefore, we have also implemented standard statistics of Rasch analyses to do pre and post-analyses of this automatic procedure's output. These features are discussed in Section 4. Also, in Section 4, the corresponding implementation in R (R Core Team 2021) is described using a real-world dataset. Finally, Section 5 summarizes our work and discusses some aspects related to the criteria and to the **autoRasch** package.

2. The Models

For the semi-automated procedure, we have implemented four models that generalize the Rasch model, i.e., the partial credit model (PCM) (Masters 1982), the generalized partial credit model (GPCM) (Muraki 1992), the partial credit model with differential item functioning (PCM-DIF) (Wijayanto *et al.* 2022), and the generalized partial credit model with differential item functioning (GPCM-DIF) (Wijayanto *et al.* 2022).

2.1. Model Expression

We first describe the most general form of the model, the GPCM-DIF. Having modeled both the discrimination and the differential functioning of items, GPCM-DIF contains the most parameters. The responses are represented as a data matrix X whose rows represent subjects and whose columns represent items. The GPCM-DIF models the general form of responses which are recorded into two or more ordered categories, $x \in \{0, 1, \ldots, m_i\}$ where $m_i + 1$ is the number of categories. According to the GPCM-DIF, the probability of subject n giving response x to item i reads

$$P(X_{ni} = x|\theta, \beta, \alpha, \delta) = \frac{\exp\left[\alpha_i \sum_{j=1}^{x}(\theta_n - (\beta_{ij} - \sum_{f=1}^{m_f} \delta_{if}\kappa_{nf}))\right]}{1 + \sum_{k=1}^{m_i} \exp\left[\alpha_i \sum_{j=1}^{k}(\theta_n - (\beta_{ij} - \sum_{f=1}^{m_f} \delta_{if}\kappa_{nf}))\right]}, \quad (1)$$

for $x > 0$, and

$$P(X_{ni} = 0|\theta, \beta, \alpha, \delta) = \frac{1}{1 + \sum_{k=1}^{m_i} \exp\left[\alpha_i \sum_{j=1}^{k} (\theta_n - (\beta_{ij} - \sum_{f=1}^{m_f} \delta_{if} \kappa_{nf}))\right]} . \quad (2)$$

Here, θ_n is the ability parameter, β_{ij} represents the difficulty (threshold) parameter of category j on item i, α_i is the item discrimination parameter, and δ_{if} is the DIF parameter that models the difference in the difficulty of item i for subjects from different groups. The binary matrix κ_{nf} maps subject n to group $f \in \{1, 2, \ldots, m_f\}$, where m_f denotes the number of potential DIF-inducing covariates: $\kappa_{nf} = 1$ if respondent n is a member of group f and $\kappa_{nf} = 0$ otherwise.

The GPCM-DIF is equivalent to the GPCM (Muraki 1992) if all δ_{if} are zero, corresponding to the case without DIF. In case all α_i are zero, we have the partial credit model with DIF (PCM-DIF) (Wijayanto *et al.* 2022). When both δ_{if} and α_i are all zero, the GPCM-DIF simplifies to the partial credit model (PCM) (Masters 1982). For binary responses, the PCM and GPCM are equivalent to the Rasch model and to the two probability logistic (2-PL) model, respectively (Rasch 1966; Lord and Novick 1968).

2.2. Penalized Joint Maximum Likelihood Estimation

Although we share the same model with the **GPCMlasso** package (Schauberger and Mair 2019), our **autoRasch** package requires a different estimation method: instead of marginal maximum likelihood estimation (MMLE) as in (Schauberger and Mair 2019), we use penalized joint maximum likelihood estimation (PJMLE) by adding penalty terms to the log likelihood.

Given the observed responses x_{ni}, we define the log likelihood for a particular set of items $\mathcal{S} \subseteq \{1, \ldots, P\}$ as

$$L_{\mathcal{S}}(\theta, \beta, \alpha, \delta) = \sum_{i \in \mathcal{S}} \sum_{n=1}^{N} \log P(X = X_{ni}|\theta, \beta, \alpha, \delta) , \quad (3)$$

with $P(X = X_{ni}|\theta, \beta, \alpha, \delta)$ as in Equations (1) and (2).

Standard JMLE is known for having difficulties in estimating extreme scores (Linacre 2004). These extreme scores tend to result in very large estimated values (either positive or negative). Therefore, we propose to use Tikhonov (L_2) regularization in order to bound the estimated value of θ_n. Furthermore, to avoid obtaining negative discrimination parameters, we use the parametrization $\alpha_i = \exp^{\gamma_i}$. Additionally, to drive the GPCM-DIF toward the standard Rasch model, we add Tikhonov regularization to the natural logarithm of α_i to drive it to one and an L_1 penalty to drive δ_i to zero.

Adding these regularizations to (3) we obtain

$$F_S(\theta, \beta, \alpha, \delta) = L_S(\theta, \beta, \alpha, \delta) - \lambda_\theta \sum_{n=1}^{N} \theta_n^2 - \lambda_\alpha \sum_{i \in S} (\ln \alpha_i)^2 - \lambda_\delta \sum_{i \in S} \sum_{f=1}^{m_f} |\delta_{if}| . \quad (4)$$

In the absence of the DIF effect, the log likelihood in (4) simplifies to

$$F_S(\theta, \beta, \alpha) = L_S(\theta, \beta, \alpha) - \lambda_\theta \sum_{n=1}^{N} \theta_n^2 - \lambda_\alpha \sum_{i \in S} (\ln \alpha_i)^2 . \quad (5)$$

2.3. Optimization Method

To optimize the log likelihood in (5), we make use of quasi-Newton methods implemented in `optim()`. However, to obtain a stable solution for (4), we resort to a two-level coordinate descent method, described in Algorithm 1. The outer loop coordinate descent aims to maximize the log likelihood and to estimate the GPCM parameters (θ, β, and α), while fixing the δ values. The inner loop coordinate descent treats the δ parameters as separate coordinates and estimates them separately, while fixing the values of the GPCM parameters.

Algorithm 1 Pseudocode for two-level coordinate descent.

procedure CoordinateDescent
    $iteration \leftarrow 0$
    $L_{(t)} \leftarrow L_{\mathcal{D}}(\theta_{init}, \beta_{init}, \alpha_{init}, \delta_{init})$
    $L_{(t-1)} \leftarrow L_{(t)} + (2 \times \eta)$
    while $(|L_{(t)} - L_{(t-1)}| > \eta)$ OR $(iteration < max.iter)$ **do** ▷ Outer loop
        $L_{(t-1)} \leftarrow L_{(t)}$
        $[\hat{\theta}, \hat{\beta}, \hat{\alpha}] \leftarrow \text{JMLE}(\mathcal{D})$
        for $j := 1$ to n_δ **do** ▷ Inner loop
            $[\hat{\delta}_j] \leftarrow \text{MLE}(\mathcal{D}, \hat{\theta}, \hat{\beta}, \hat{\alpha}, \hat{\delta}_{[\text{other than j}]})$
        end for
        $L_{(t)} \leftarrow L_{\mathcal{D}}(\hat{\theta}, \hat{\beta}, \hat{\alpha}, \hat{\delta})$
        $iteration \leftarrow iteration + 1$
    end while
end procedure

3. Semi-Automated Procedure

3.1. The Criteria

In (Wijayanto *et al.* 2021) and (Wijayanto *et al.* 2022), we introduced novel criteria to evaluate the quality of an instrument from a given original survey. In summary, when performing a standard Rasch analysis for a given original survey $\mathcal{S}$, items that do not satisfy certain criteria are moved to the excluded set ($\mathcal{S}_{\text{out}}$). Based on the remaining items, gathered in the included set ($\mathcal{S}_{\text{in}}$), we then construct the most representative scale for the subjects. In our package, we do the same when estimating the GPCM-DIF parameters by optimizing (4) with $S = \mathcal{S}_{\text{in}}$:

$$\{\hat{\theta}_{\mathcal{S}_{\text{in}}}, \hat{\beta}_{\mathcal{S}_{\text{in}}}, \hat{\alpha}_{\mathcal{S}_{\text{in}}}, \hat{\delta}_{\mathcal{S}_{\text{in}}}\} = \underset{\{\theta,\beta,\alpha,\delta\}}{\text{argmax}}\, L_{\mathcal{S}_{\text{in}}}(\theta, \beta, \alpha, \delta) - \lambda_\theta \sum_{n=1}^{N} \theta_n^2 - \lambda_{\text{in}} \sum_{i \in \mathcal{S}_{\text{in}}} (\ln \alpha_i)^2 - \lambda_\delta \sum_{i \in \mathcal{S}_{\text{in}}} \sum_{f=1}^{m_f} |\delta_{if}| \,.$$

We refer to the log likelihood of these fitted parameters on the included itemset as the in-questionnaire log likelihood with DIF:

$$\text{IQ-LL-DIF}(\mathcal{S}_{\text{in}}) = L_{\mathcal{S}_{\text{in}}}(\hat{\theta}_{\mathcal{S}_{\text{in}}}, \hat{\beta}_{\mathcal{S}_{\text{in}}}, \hat{\alpha}_{\mathcal{S}_{\text{in}}}, \hat{\delta}_{\mathcal{S}_{\text{in}}}) \,.$$

By construction, the excluded items in $\mathcal{S}_{\text{out}}$ no longer have an effect on the scale. Nevertheless, assuming they have not been considered in the initial survey without a reason, we still would

like the estimated scale to also have a decent fit to these excluded items. To estimate the quality of this fit, we now clamp $\theta_{\mathcal{S}_{\text{out}}}$ to $\hat{\theta}_{\mathcal{S}_{\text{in}}}$ and estimate the other parameters of the excluded items by optimizing the log-likelihood

$$\{\hat{\beta}_{\mathcal{S}_{\text{out}}}, \hat{\alpha}_{\mathcal{S}_{\text{out}}}, \hat{\delta}_{\mathcal{S}_{\text{out}}}\} = \underset{\{\beta,\alpha\}}{\text{argmax}}\, L_{\mathcal{S}_{\text{out}}}(\hat{\theta}_{\mathcal{S}_{\text{in}}}, \beta, \alpha, \delta) - \lambda_{\text{out}} \sum_{i \in \mathcal{S}_{\text{out}}} (\ln \alpha_i)^2 - \lambda_\delta \sum_{i \in \mathcal{S}_{\text{out}}} \sum_{f=1}^{m_f} |\delta_{if}| \,.$$

We refer to the resulting

$$\text{OQ-LL-DIF}(\mathcal{S}_{\text{out}}) = L_{\mathcal{S}_{\text{out}}}(\hat{\theta}_{\mathcal{S}_{\text{in}}}, \hat{\beta}_{\mathcal{S}_{\text{out}}}, \hat{\alpha}_{\mathcal{S}_{\text{out}}}, \hat{\delta}_{\mathcal{S}_{\text{out}}})$$

as the out-of-questionnaire log likelihood with DIF. Taking the total of both log likelihoods leads us to one of our criteria:

$$\text{IPOQ-LL-DIF}(\mathcal{S}_{\text{in}}, \mathcal{S}_{\text{out}}) = \text{IQ-LL-DIF}(\mathcal{S}_{\text{in}}) + \text{OQ-LL-DIF}(\mathcal{S}_{\text{out}}) \,.$$

In absence of differential item functioning, we set $\delta = 0$ and only estimate θ, β, and γ. This gives the simpler criterion IPOQ-LL (Wijayanto *et al.* 2021). Since both criteria are meant to score the quality of an itemset for constructing a scale based on the given responses, the higher these scores the better.

3.2. Searching for the Optimal Itemset

For any choice of included itemset $\mathcal{S}_{\text{in}}$ and corresponding excluded itemset $\mathcal{S}_{\text{out}}$, we can compute the IPOQ-LL or IPOQ-LL-DIF. To search for an optimal itemset, **autoRasch** makes use of stepwise selection. Stepwise selection is a mix between backward elimination (eliminating variables one-by-one) and forward selection (adding variables one-by-one). The step-by-step procedure of the stepwise selection is presented in Algorithm 2. Stepwise selection starts with backward elimination on a full itemset to eliminate the worst item. OneStepBackwardElimination($\mathcal{S}_{\text{in}}$) in line 5 considers all possible itemsets with one item fewer and returns the highest in-plus-out-of-questionnaire log likelihood as well as the itemset that leads to this maximum. In lines 8 and 11, OneStepForwardSelection($\mathcal{S}_{\text{in}}$) does the same, but only considers itemsets that can be constructed by adding one additional item to $\mathcal{S}_{\text{in}}$. In practice, stepwise selection provides a good trade-off between very efficient greedy methods like backward elimination and forward selection and much more computationally demanding procedures such as best subset selection, which considers all possible combinations of items.

4. The autoRasch Package and Application Examples

4.1. Implementation in autoRasch

The **autoRasch** package fits the models presented in Section 2.1 using penalized joint maximum likelihood estimation (PJMLE) as formulated in Section 2.2. We have implemented four fitting functions (i.e., `gpcm_dif()`, `gpcm()`, `pcm_dif()`, and `pcm()`) to estimate the parameters of the GPCM-DIF, GPCM, PCM-DIF, and PCM, respectively. The Rasch model, a special case of the PCM, can be fit using `pcm()` and the 2-PL model, a special case of the GPCM, can be fit using `gpcm()`. Each of these fitting functions returns an object of a class

Algorithm 2 Pseudocode for stepwise selection (Wijayanto *et al.* 2021).

procedure StepwiseSelection
  $\mathbf{M} = -\infty$ ▷ Initialize the list of highest scores for each $|\mathcal{S}_{\text{in}}|$
  $\mathcal{S}_{\text{in}} = \{1, \ldots, P\}$
  while $|\mathcal{S}_{\text{in}}| > 0$ **do**
    $[M_{\text{highest}}, \mathcal{S}_{\text{in}}] \leftarrow \text{OneStepBackwardElimination}(\mathcal{S}_{\text{in}})$
    if $M_{\text{highest}} > M_{|\mathcal{S}_{\text{in}}|}$ **then**
      $M_{|\mathcal{S}_{\text{in}}|} = M_{\text{highest}}$
      $[M_{\text{highest}}, \mathcal{S}_{\text{in}}] \leftarrow \text{OneStepForwardSelection}(\mathcal{S}_{\text{in}})$
      while $|\mathcal{S}_{\text{in}}| < P$ and $M_{\text{highest}} > M_{|\mathcal{S}_{\text{in}}|}$ **do**
        $M_{|\mathcal{S}_{\text{in}}|} = M_{\text{highest}}$
        $[M_{\text{highest}}, \mathcal{S}_{\text{in}}] \leftarrow \text{OneStepForwardSelection}(\mathcal{S}_{\text{in}})$
      end while
    end if
  end while
end procedure

named after the function (e.g., `pcm()` returns a `pcm` object, `pcm_dif()` returns a `pcm_dif` object, etc.), for which the methods `print()` and `summary()` are available.

The semi-automated procedure searches automatically for the maximum score of either the IPOQ-LL or IPOQ-LL-DIF (see Section 3.1). These criteria can be computed using `compute_score()` for a single itemset and `compute_scores()` for multiple itemsets at once in parallel. The maximum of the criteria can be obtained using `stepwise_search()`, which implements Algorithm 2. The result of the search procedure can be plotted using `plot_search()`.

Having obtained an itemset with the highest score, one could always reevaluate its performance using standard statistics of Rasch analyses or even perform an additional manual Rasch analysis starting from this itemset. The **autoRasch** package also implements some standard statistics (e.g., item goodness-of-fit, correlation of the residual, and separation reliability). The unweighted mean square (OutfitMnSq), weighted mean square (InfitMnSq), standardized unweighted mean square (OutfitZSTD), and standardized weighted mean square (InfitZSTD) are provided to measure how well an item fits to a model (Wright and Masters 1982, 1990). These statistics can be computed using `fitStats()`. As for the local dependency, we can compute the correlation of the residual using `residCor()`. To measure the reliability of the itemset, one can use `checkRel()`, which implements the person/item separation reliability statistics (Wright and Masters 1982). Additionally, the package is also equipped with graphical tools, e.g., person item map (`plot_PImap()`) and item characteristic curves (`plot_ICC()`).

4.2. Application in the Interdisciplinary Education Perception Scale

In this section we will demonstrate how to perform a semi-automated Rasch analysis using **autoRasch**. We consider a real-world dataset from the interdisciplinary education perception scale (IEPS) (Vaughan 2019). The IEPS dataset consists of responses from 319 subjects to 18 items and four potential DIF-inducing covariates that contain subjects' information (i.e., university, year level, age group, and gender). For this demo, we only used two covariates:

gender and university. After rescoring some items to eliminate disordered thresholds, the data is then analyzed using semi-automated Rasch analysis as described in (Vaughan 2019).

```
R> library("autoRasch")
R> head(ieps)
```

```
Uni Sex V1 V2 V3 V4 V5 V6 V7 V8 V9 V10 V11 V12 V13 V14 V15 V16 V17 V18
[1,]   1   1  4  3  3  3  2  2  2  3  3   2   2   3   2   2   2   1   4   3
[2,]   1   1  4  3  2  4  1  2  2  2  3   1   3   2   2   3   1   1   2   3
[3,]   1   1  4  4  4  4  3  3  3  2  4   2   3   4   3   4   3   3   4   4
[4,]   1   1  4  3  3  4  3  3  3  2  4   2   2   3   2   3   2   2   4   4
[5,]   1   2  4  4  4  5  3  3  3  1  5   1   3   4   3   4   3   3   4   3
[6,]   1   1  4  3  2  4  2  2  2  2  4   2   3   2   3   4   2   1   3   3
```

```
R> GM_ieps <- createGroup(ieps[,1:2])
R> iepsWorkData <- ieps[,3:20]
R> searchResult <- stepwise_search(X = iepsWorkData, criterion = "ipoqlldif",
+  groups_map = GM_ieps, cores = 20)
```

First, the **autoRasch** package needs to be loaded with `library()`. The responses from n subjects to i items of the original survey are stored as a `matrix` or `data.frame`. To preview the dataset, `head()` can be used. Here columns 1 and 2 consist of respondents' background information and the responses are in columns 3 to 20. We then store the responses to `iepsWorkData`. Passing in these responses, we use `stepwise_search()` to find an optimal itemset in a stepwise manner based on the chosen criterion; `criterion = "ipoqlldif"` to take DIF effects into account and `criterion = "ipoqll"` otherwise. The `ipoqlldif` requires `groups_map` parameters, an $n \times m_f$ binary matrix that maps n subjects into m_f focal groups based on the subjects' background information for estimating the DIF effect. To accommodate this mapping process, `createGroup()` can be used to group the subjects automatically. Additionally, to speed up the search, `stepwise_search()` allows for parallelization by setting the number of cores through `cores`.

```
R> summary(searchResult)
```

```
Maximum IPOQ-LL-DIF score is obtained with 14 items in the included set.
Items no.:  1,2,4,5,7,9,10,12,13,14,15,16,17,18

IQ-LL-DIF OQ-LL-DIF IPOQ-LL-DIF
-4180.271 -1589.002   -5769.273
```

To summarize the search result, `summary()` will return the set of items that scores the highest, accompanied by the corresponding scores.

```
R> plot_search(search_result, type = "l", ylim = c(-5600,-5100),
+  cex.lab = 1.3, cex = 1.3, cex.axis = 1.1)
```

Additionally, utilizing `plot()` we can track the highest IPOQ-LL-DIF score for every $|\mathcal{S}_{\text{in}}|$. In Figure 1, starting from the full set, items are removed sequentially and the scores first increase and then decrease with a peak at $|\mathcal{S}_{\text{in}}| = 14$. The IPOQ-LL-DIF and IPOQ-LL criteria score how well the responses fit the model, where the highest value is to be preferred. In this case, IPOQ-LL-DIF scores from $|\mathcal{S}_{\text{in}}| = 16$ to $|\mathcal{S}_{\text{in}}| = 8$ are very similar, suggesting that a shorter instrument can also be taken under consideration.

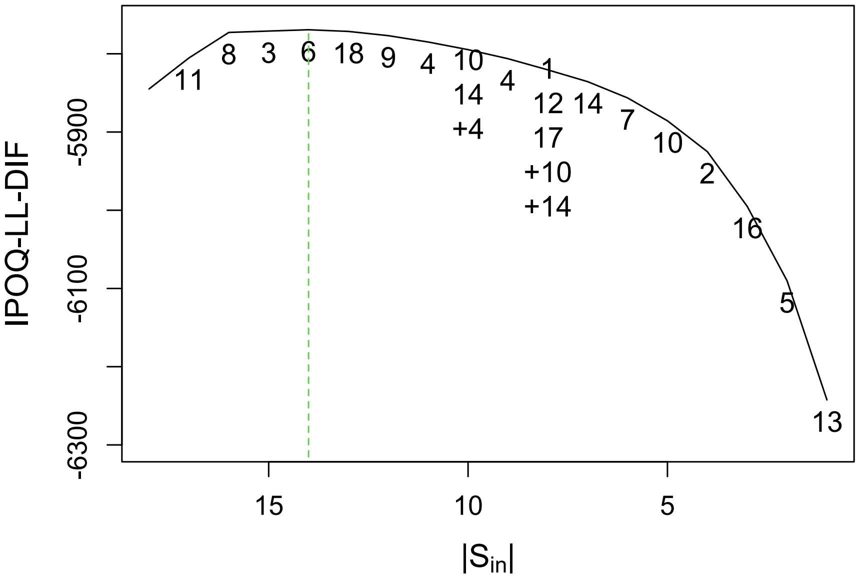

Figure 1: The `plot()` of the highest IPOQ-LL-DIF for various $|\mathcal{S}_{\text{in}}|$. The numbers on the plot show in which order the items are removed, e.g., $|\mathcal{S}_{\text{in}}| = 8$ was formed after removing item$_1$, item$_{12}$, and item$_{17}$, but reintroducing item$_{10}$ and item$_{14}$ which had been removed before. The dashed green line locates the maximum value of IPOQ-LL-DIF.

Having obtained an itemset using the automated method, one could always reevaluate its performance using the statistics of standard Rasch analyses. If needed, a Rasch analysis can be initiated manually from the obtained itemset. To compute the parameter estimates of a partial credit model, one may use `pcm()`, which implements penalized joint maximum likelihood estimation as described in Section 2.2. In the presence of DIF, one should use `pcm_dif()` to incorporate the effect of DIF into the estimation procedure.

```
R> pcmResult <- pcm_dif(iepsWorkData[,c(1,2,4,5,7,9,10,12,13,14,15,16,17,18)])
R> summary(pcmResult)
R> plot_PImap(pcmResult)
```

```
The estimated ability scores (theta):
[1]  0.21 -0.47  3.22  1.61  3.26  0.78 -0.59  0.81 -0.78  0.98  4.70 -1.97
[13]  1.18  0.03  0.21 -0.89  0.43  0.25  0.25  1.01  0.25  1.01 -0.14  0.39
[25]  0.78  0.78 -0.11 -1.68 -1.07  0.98 -0.14 -1.68  0.03  0.21 -0.14 -0.78
[37]  0.25 -2.09 -0.47 -0.78 -1.47 -0.44  1.87  0.25  0.07 -0.14  0.78  1.42
[49]  1.64  0.43  0.25  0.03 -0.78  0.21 -0.27  2.11 -0.89 -0.27  2.36 -1.68
[61]  0.62  0.58  0.98 -1.07 -0.59  0.21  1.64 -0.44 -1.43 -0.59 -1.03 -0.44
```

```
[73] -1.30  2.11  0.25 -0.74  1.87 -0.11  0.43 -0.44  0.62  1.42 -0.14  0.98
[85]  0.98 -0.44  0.78  0.03  2.11 -0.63 -0.78 -1.07 -0.11  0.39 -1.68  1.39
[97] -1.47  0.62 -1.68 -0.44  0.62  4.13 -0.59  0.00 -0.93 -0.31 -1.21  0.07
[109]  0.78  0.78  0.81 -1.17 -1.43 -1.34 -1.07  0.81 -0.31  0.03 -0.78 -2.41
[121] -1.47  0.62 -0.47 -1.07  0.62  0.43 -0.27 -0.78 -0.14 -1.68 -0.74 -2.92
[133] -0.47 -2.33 -1.56 -1.34 -1.34 -0.14 -0.59  0.03 -0.74 -1.03 -2.41  0.62
[145]  0.71  0.21 -1.21  0.62 -0.89 -0.27 -1.03 -0.27 -1.17 -0.44  0.78 -1.21
[157] -0.93  0.78  0.78 -0.44  0.03 -1.07 -0.78 -1.21 -1.07  0.58  0.39 -0.59
[169]  1.42  0.58  1.39  1.42 -0.44 -1.56  0.03  0.21 -0.93  0.07 -1.68 -0.31
[181]  0.62 -0.31  0.81  0.62  1.42 -1.43  1.64 -2.96 -1.72  1.21  1.21 -0.59
[193]  0.43  0.81 -0.59  0.07  2.36 -0.11 -0.11 -0.74 -1.30 -1.34  2.11 -1.21
[205]  0.07  3.26  0.62  1.42 -0.14 -1.43 -1.72  0.25 -0.74  0.81  0.07 -0.11
[217]  0.81  0.43 -0.63 -2.17  1.01  2.11  0.78  0.39  1.64  1.21 -0.44 -0.31
[229] -1.21 -0.63 -0.14 -1.47  0.78 -0.63  1.84 -0.27  0.58 -0.44 -0.44  0.25
[241]  3.22 -0.27 -0.11  1.64 -0.27  5.77  1.87  0.25  0.98 -0.59  1.01 -0.31
[253] -0.11  0.21  0.58 -0.63  1.18 -0.59 -0.44 -1.43  0.62 -0.89  1.64  0.07
[265]  0.28 -1.39 -0.40 -1.52  0.81  0.28 -1.69 -1.81  0.24 -0.44 -0.11 -0.89
[277]  0.62  0.28 -1.17 -1.03  0.10 -1.89  0.10 -1.26 -0.56  1.21  1.64 -0.59
[289]  0.65  0.28 -0.07 -0.44 -0.89  0.28 -0.07  0.65 -1.31 -0.89 -0.59  2.11
[301]  0.84 -1.44 -1.69  0.65  0.84 -0.40  1.67  0.62 -0.11 -0.40  1.45 -0.71
[313] -0.75 -0.24 -0.59 -0.24  1.04  2.96

The highest ability score:  5.77
The lowest ability score:  -2.96
Respondent(s) no.  310  are excluded due to missing (incomplete) background information.

The estimated difficulty scores (beta):
Th_1  Th_2  Th_3 Th_4 Th_5 Item Loc.
V1  -3.79 -2.81 -2.22 0.59   NA     -2.06
V2  -4.66 -2.45 -0.63 1.70   NA     -1.51
V4  -4.62 -2.04 -1.61 0.66 3.37     -0.85
V5  -3.54 -1.45  1.33   NA   NA     -1.22
V7  -3.24 -1.96  1.26   NA   NA     -1.31
V9  -2.98 -2.21 -1.30 0.95 4.26     -0.26
V10 -3.02 -0.77  2.47   NA   NA     -0.44
V12 -2.58 -2.05 -0.07 3.06   NA     -0.41
V13 -2.35 -1.41  1.43   NA   NA     -0.78
V14 -4.21 -2.86 -1.04 1.69   NA     -1.60
V15 -2.76 -0.43  2.26   NA   NA     -0.31
V16 -2.16  0.02  2.89   NA   NA      0.25
V17 -3.42 -3.55 -1.72 1.18   NA     -1.88 *
V18 -4.72 -2.03 -1.60 0.25 2.66     -1.09

The most difficult item:  V16
The easiest item:  V1
There are 1 items which have disordered thresholds.
'*' Item has disordered thresholds.
```

```
The estimated DIF effects (gap of difficulties) (delta):
Univ  Sex
V2       0.07
V4       0.03
V9       0.32
V18 0.35
```

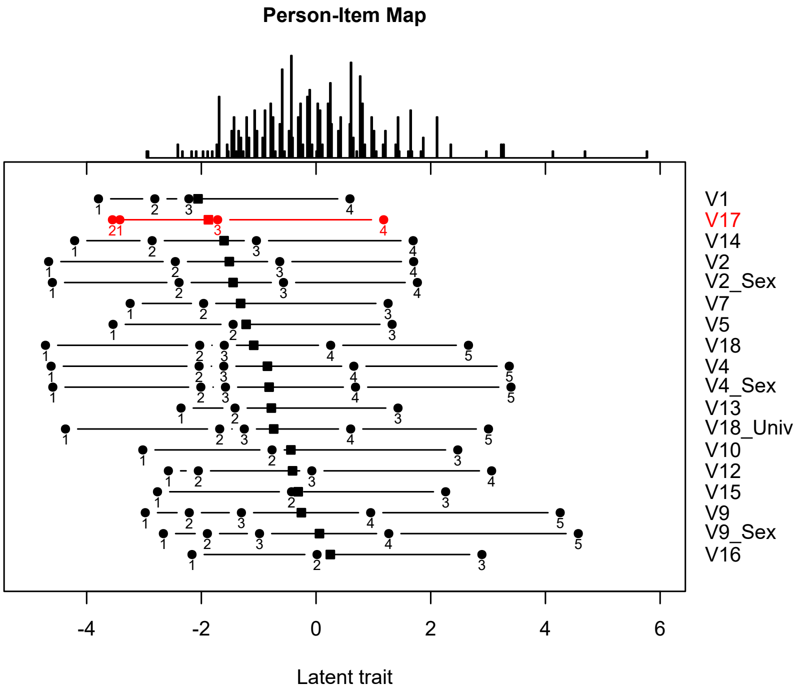

Figure 2: The person-item map of 14 items that results in the highest IPOQ-LL-DIF. The `_Name` appended to a variable indicates the name of the covariate that induced DIF in a particular item. For example, `V9` maps the difficulty of item `V9` for the reference group whereas `V9_sex` maps the difficulty for the focal group when gender is the DIF-inducing covariate.

```
R> plot_ICC(pcmResult, itemno = 13)
```

The fitting function for the PCM-DIF model, `pcm_dif()`, estimates all parameters including `delta`, which models the difference in difficulty between groups for each item. The `pcm_dif()`, however, can only estimate abilities of subjects with background information and omit the subjects otherwise. Having these estimates, the estimated parameters can be plotted as a person-item map using `plot_PImap()`. On the person-item map, item_{17} is highlighted in red, which indicates a disordered threshold. If needed, the item characteristic curve (ICC) of item_{17} can be plotted using `plot_ICC()` as in Figure 3. The items' goodness-of-fit can be examined using the standard statistics of Rasch analyses, namely unweighted mean square

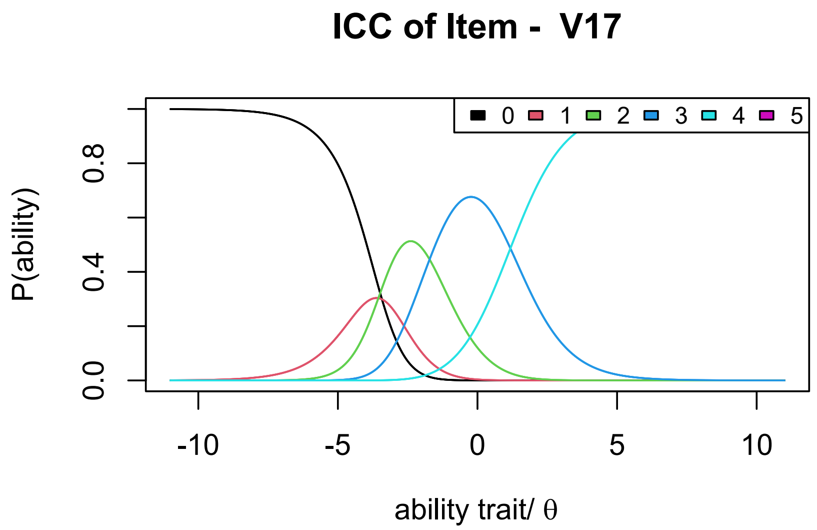

Figure 3: The item characteristic curve (ICC) of item_{17}.

(OutfitMnSq), weighted mean square (InfitMnSq), standardized unweighted mean square (OutfitZSTD), and standardized unweighted mean square (InfitZSTD) (Wright and Masters 1982, 1990). These statistics are implemented in `fitStats()` for objects of either class `pcm` or `pcm_dif`.

```
R> pcmFit <- fitStats(pcmResult)
R> itemfit(pcmFit)

Item Fit Statistics:

OutfitMnSq InfitMnSq OutfitZSTD InfitZSTD alpha
V1         0.96       0.99       -0.40      -0.13  1.05
V2         1.01       1.01        0.18       0.16  0.98
V4         1.13       1.11        1.54       1.32  0.86
V5         0.88       0.89       -1.49      -1.45  1.15
V7         0.88       0.91       -1.29      -1.09  1.13
V9         1.18       1.14        2.10       1.59  0.85
V10        0.92       0.90       -1.00      -1.27  1.12
V12        1.09       1.08        1.06       0.91  0.95
V13        0.91       0.92       -0.98      -0.93  1.12
V14        0.86       0.90       -1.66      -1.22  1.16
V15        0.79       0.80       -2.85      -2.78  1.26
V16        0.90       0.90       -1.37      -1.34  1.12
V17        0.92       0.95       -0.84      -0.54  1.06
V18        1.24       1.22        2.75       2.56  0.81

R> par(pty = "s")
```

```
R> plot_fitStats(pcmFit, type = "n", useName = TRUE,
+ xlim = c(0.75,1.35), ylim = c(0.75,1.35))
```

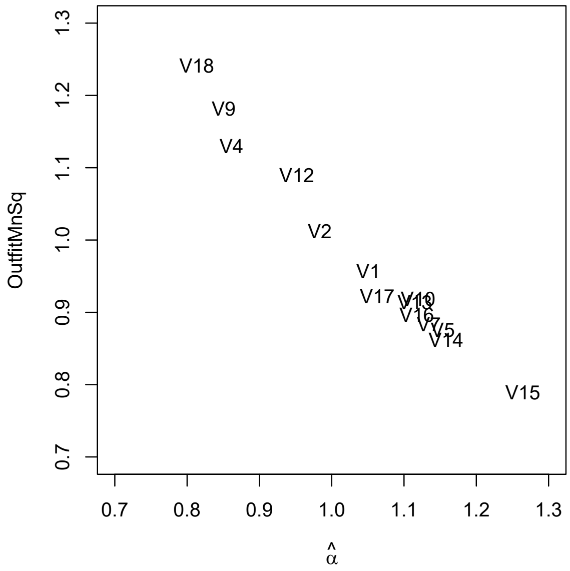

Figure 4: Estimated alpha ($\hat{\alpha}$) values against Outfit mean square (OutfitMnSq) which is plotted using `plot_fitStats()`.

The items' goodness-of-fit statistics can be summarized using `itemfit()`. Additionally, `fitStats()` also returns the corresponding discrimination parameters (`alpha`) that are obtained after fitting the data to either the GPCM or GPCM-DIF for objects of class `pcm` or `pcm_dif`, respectively. Normally, these five values are well correlated and any deviation implies the need to check the values, e.g., a high `OutfitMnSq` value because of surprising responses from a single subject (see Wijayanto *et al.* (2021)). The correlation can be visualized using `plot_fitStats()` as shown in Figure 4.

The local item dependency is another issue that needs to be checked since it has been shown to have a strong relation with unidimensionality. To check the local dependency status of an itemset, **autoRasch** has been equipped with `corResid()` so as to compute the correlation of the residual for evaluating the local dependency status of an itemset.

```
R> pcmLD <- corResid(pcmFit)
R> summary(pcmLD)

Correlation of the standardized residual:

There are 2 pair(s) of variable(s) with correlation >= 0.3
1. V9 and V4
2. V7 and V5
```

Another potential concern is the reliability of the estimates in the Rasch analysis. The typical statistic used is the separation reliability which is accessed via `checkRel()`. This function returns both the index and reliability of the separation of both person and item.

```
R> pcmRel <- checkRel(pcmResult)
R> summary(pcmRel)

Person  Item
Reliability Index        2.60 36.86
Separation Reliability   0.87  1.00
RMSSE                    0.43  0.33
```

5. Summary and discussion

In this article, we have presented the **autoRasch** package, a tool for performing Rasch analyses in a semi-automated way. This approach has been shown to provide comparable, statistically indistinguishable results compared to standard Rasch analyses (see Wijayanto *et al.* (2021) and Wijayanto *et al.* (2022)).

Two criteria have been developed to be used in different circumstances. The IPOQ-LL-DIF is used to include the effect of the differential functioning in items. The IPOQ-LL disregards this DIF effect, making its computation faster. To select the items that make up the final instrument, we chose to implement a stepwise-search optimization method. Other optimization methods could be implemented in the future.

The **autoRasch** package can be used to automatically arrive at an instrument, without manual interventions. As argued before, we do recommend to reevaluate the quality of the final set based on one's expertise. Alternatively, **autoRasch** can be used to quickly arrive at an initial set of items that are used as a starting point for a standard Rasch analysis. Accompanied by one's expertise and guided by some typical statistics in Rasch analyses, this **autoRasch** package has the potential to find high-quality items with a lower cost in effort and time. **autoRasch**'s functions are designed to be as user-friendly as possible without the need for complicated settings.

References

Linacre JM (2004). "Rasch model estimation: further topics." *Journal of applied measurement*, **5**(1), 95–110.

Lord FM, Novick MR (1968). *Statistical Theories of Mental Test Scores*. Information Age Publishing Inc. ISBN 9781593119348.

Masters GN (1982). "A rasch model for partial credit scoring." *Psychometrika*, **47**(2), 149–174. ISSN 00333123. doi:10.1007/BF02296272.

Muraki E (1992). "A generalized partial credit model: Application of an EM algorithm." *Applied Psychological Measurement*, **16**(2). doi:10.1177/014662169201600206.

R Core Team (2021). *R: A Language and Environment for Statistical Computing*. R Foundation for Statistical Computing, Vienna, Austria. URL https://www.R-project.org/.

Rasch GG (1966). "An item analysis which takes individual differences into account." *British Journal of Mathematical and Statistical Psychology*, **19**, 49–57.

Schauberger G, Mair P (2019). "A regularization approach for the detection of differential item functioning in generalized partial credit models." *Behavior Research Methods*, pp. 1–16. doi:10.3758/s13428-019-01224-2.

Vaughan B (2019). "Measurement Properties of the Interdisciplinary Education Perception Scale in an Australian Allied Health Student Cohort." *Health Professions Education*, **5**(3), 275–280. doi:10.1016/j.hpe.2018.07.005.

Wijayanto F, Bucur IG, Mul K, Groot P, van Engelen BG, Heskes T (2022). "Semi-automated Rasch analysis with differential item functioning." *PsyArxiv preprint*. URL https://psyarxiv.com/erxuh.

Wijayanto F, Mul K, Groot P, van Engelen BG, Heskes T (2021). "Semi-automated Rasch analysis using in-plus-out-of-questionnaire log likelihood." *British Journal of Mathematical and Statistical Psychology*, **74**(2), 313–339. doi:10.1111/bmsp.12218.

Wright BD, Masters GN (1982). *Rating Scale Analysis*. MESA Press, Chicago.

Wright BD, Masters GN (1990). "Computation of outfit and infit statistics." *Rasch Measurement Transactions*, **3**(4), 84–85. URL https://www.rasch.org/rmt/rmt34e.htm.

Affiliation:

Feri Wijayanto[1,2], Ioan Gabriel Bucur[1], Perry Groot[1], Tom Heskes[1]
[1]Institute for Computing and Information Sciences, Radboud University Nijmegen
Toernooiveld 212, 6525 EC Nijmegen, The Netherlands
E-mail: f.wijayanto, ig.bucur, perry.groot, tom.heskes@science.ru.nl

[2]Department of Informatics, Universitas Islam Indonesia
Jl. Kaliurang Km. 14., Sleman, DIY, Indonesia
E-mail: feri.wijayanto@uii.ac.id
